# The Westermo network traffic data set

**DOI:** 10.1016/j.dib.2023.109512

**Published:** 2023-08-24

**Authors:** Per Erik Strandberg, David Söderman, Alireza Dehlaghi-Ghadim, Miguel Leon, Tijana Markovic, Sasikumar Punnekkat, Mahshid Helali Moghadam, David Buffoni

**Affiliations:** a Westermo Network Technologies AB, Västerås, Sweden; b RISE Research Institutes of Sweden, Västerås, Sweden; c Mälardalen University, Västerås, Sweden; d Tietoevry, Stockholm, Sweden

**Keywords:** Industrial communication system, Cyber-physical systems, Network intrusion detection, Distributed artificial intelligence

## Abstract

There is a growing body of knowledge on network intrusion detection, and several open data sets with network traffic and cyber-security threats have been released in the past decades. However, many data sets have aged, were not collected in a contemporary industrial communication system, or do not easily support research focusing on distributed anomaly detection. This paper presents the Westermo network traffic data set, 1.8 million network packets recorded in over 90 minutes in a network built up of twelve hardware devices. In addition to the raw data in PCAP format, the data set also contains pre-processed data in the form of network flows in CSV files. This data set can support the research community for topics such as intrusion detection, anomaly detection, misconfiguration detection, distributed or federated artificial intelligence, and attack classification. In particular, we aim to use the data set to continue work on resource-constrained distributed artificial intelligence in edge devices. The data set contains six types of events: harmless SSH, bad SSH, misconfigured IP address, duplicated IP address, port scan, and man in the middle attack.

**Specifications Table**

**Table:** 

Subject	Computer Science
Specific subject area	Artificial Intelligence, Computer Networks and Communications, Embedded Systems
Type of data	Network packet capture (PCAP) files, and extracted network flows with features and labels as comma separated values (CSV) files
How the data were acquired	On a laptop with tcpdump (see detailed description below), while connected to a network of 12 devices running a factory simulator.
Data format	Raw as well as preprocessed.
Description of data collection	Network traffic was captured in a network topology built up of Westermo routers acting as an industrial communication network, as well as Raspberry Pi devices running the ICSSIM factory simulator (see detailed description below).
Data source location	Data was collected at Westermo Network Technologies AB, on Metallverksgatan, in Västerås, Sweden.
Data accessibility	Data is available at GitHub: https://github.com/westermo/network-traffic-dataset DOI: 10.5281/zenodo.8254402

## 1 Value of the Data

• The Westermo network traffic data set can be used for conducting research on cyber-security, in particular in the domain of Artificial Intelligence (AI) applications for network intrusion detection. A specific focus can be on different research areas within Machine Learning (ML), including application of various supervised, semi-supervised, and unsupervised ML techniques. Various ML problems can be addressed, including binary and multi-class classification, regression, clustering, and pattern recognition. This data set is very valuable to support the research in the area of distributed and federated learning since the data set was recorded on multiple locations in a distributed system. Local AI models can be deployed on clients with a meta-model created on a server. This specific dataset has three clients (left, bottom, right), and their data can be used to train local models for clients. The local models can be merged in the server and sent back to clients for further use.
• There are multiple existing data sets that are widely used in the network intrusion detection research area. The most known ones include: KDD99 [5], NSL-KDD [1], UNSW-NB15 [8], and CIC-IDS-2017 [9]. For all of those data sets, data about network packets were recorded and then preprocessed to create the features. Every entry was labeled either as normal activity or as some type of network attack. All of them were created in the simulated environment, containing normal traffic and different types of network attacks. The data set presented in this paper is created in the same manner but with some crucial differences that bring novelties compared to previous data sets:
  ○ data collection occurred in several places in the network simultaneously,
  ○ in addition to cyber security anomalies (such as port scanning), human errors (such as misconfigurations) were also included,
  ○ data was collected using an industrial control network,
  ○ preprocessed data is based on network flows instead of on individual network packets, and
  ○ two different labeling strategies were used.

## 2 Objective

The release of this data set is motivated by several factors:

1. High value to research: Realistic industrial data is frequently requested by researchers. As far as we can tell, network traffic data sets are not often collected in multiple places in the same network topology in the same experiment, which is a setup required for development of distributed/federated AI-powered network anomaly detection. Additionally, the dataset contains extra classes that have not been considered in previously published dataset. Furthermore, the dataset was collected using twelve physical devices, including industrial routers in a network topology that mirrors an industrial network.
2. Beneficence in general: Releasing the data might do well as new research, algorithms, or tools could be valuable not only for the research community or Westermo as a company but also for the industry and the general public.
3. Industry-academia relations: One often says that there is a distance between academia and industry; the release of data could hopefully render researched solutions more realistic and would thereby lower thresholds for industrial adoption of research artifacts, as well as simplify relations between academia and industry.

## 3 Data Description

**Fig. 1:**
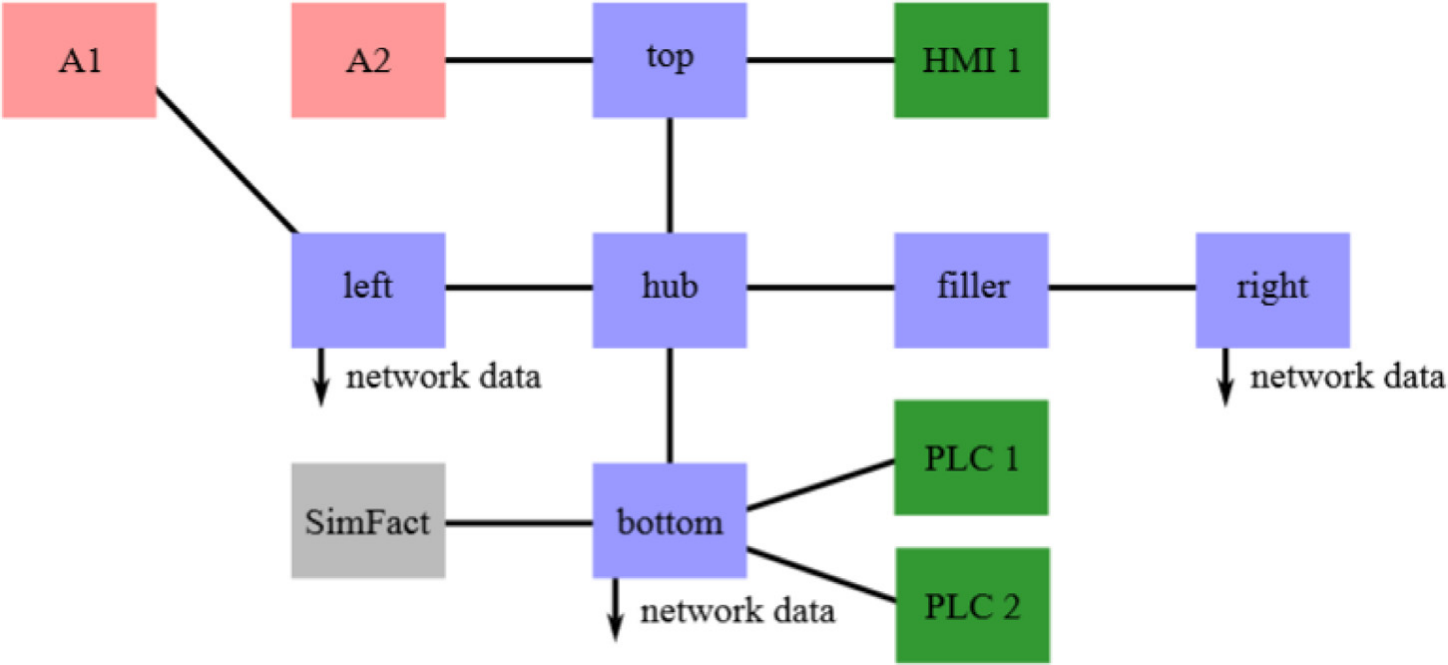
Overall network topology. Pink: A1 and A2 were used for generating anomalies. Blue: Westermo devices in the controller network. Green: Raspberry Pis running HMI or PLCs of the factory simulator. Gray: Raspberry Pi running the physical world simulated factory (SimFact). See Table 3 for details.

The network traffic was collected in a physical network topology constructed to be similar to an industrial communication network, see Figs. 1 and 3, as well as Table 3. With the Industrial Communication System Simulator (ICSSIM)^1^ [3], this network simulated a bottle filling factory: two programmable logic controllers (PLCs) interact with a water tank, conveyer belt, etc., to fill bottles one at a time, and one human-machine interface (HMI) presents the status to a human operator. For this purpose, twelve physical devices were involved: one laptop, six routers, and five Raspberry Pi (RPI) devices, see details in Table 3. The six routers acted as the industrial communication system (the network) and ran the very common rapid spanning tree protocol (RSTP) for redundancy. One of the RPIs acted as the HMI of the factory simulator, and two acted as PLCs. The fourth RPI (SimFact in Fig. 1) ran the simulator for the physical world (with water tanks, etc.), and the final RPI ran the attack toolkit from ICSSIM. Before data collection started, the system was in an operational state: the nodes in ICSSIM communicated with each other over the core network. In particular, the PLC's informed the HMI on states in the factory, e.g. water level in the simulated tank, states in valves, distances between bottles and filler, etc. In addition, RSTP sends control traffic in the idle state.

### 3.1 Raw data

The raw data consists of three PCAP files of network traffic collected with tcpdump. Each packet represents a packet going into or out of one of the recording devices: left, right, or bottom, see Fig. 1. Some packets would first go into the device and then out of it, so there are many duplicated packets in the data. The physical world and factory simulator of ICSSIM was used in the data collection, and some of this traffic is not representative of a factory. For this reason, we present two sets of PCAP files: the reduced set where the traffic needed for the physical world simulator is removed, and the extended data set where it is kept. See Table 1 for an overview of the number of packets in the PCAP files, as well as an overview of the communication protocols used.

**Table 1:** Overview of packets in data.

	Reduced						Extended					
	left		bottom		right		left		bottom		right	
	N	%	N	%	N	*%*	N	*%*	N	*%*	N	*%*
ARP	12547	*4.5*	10230	*0.7*	6183	*17.6*	12639	*4.5*	11513	*0.3*	6234	*17.7*
ICMP	26	*0.01*	-	*-*	-	*-*	26	*0.01*	4636	*0.1*	6	*0.02*
IGMP	1650	*0.6*	2875	*0.2*	438	*1.2*	1650	*0.6*	3284	*0.1*	438	*1.2*
LLDP	905	*0.3*	1086	*0.1*	543	*1.5*	905	*0.3*	1086	*0.02*	543	*1.5*
RSTP	8163	*2.9*	13608	*0.9*	5445	*15.5*	8163	*2.9*	13608	*0.3*	5445	*15.5*
TCP	256378	*91.2*	1484940	*98.1*	21740	*62.0*	256378	*91.2*	4348980	*99.2*	21740	*61.9*
UDP	1436	*0.5*	1076	*0.1*	718	*2.0*	1436	*0.5*	1076	*0.02*	718	*2.0*
Total	281105	*100.0*	1513815	*100.0*	35067	*100.0*	281197	*100.0*	4384183	*100.0*	35124	*100.0*

When network events are triggered, this is described in a log-file with timestamps and other information needed to make sense of the network traffic. One could say that this description contains information on labels of the network as a whole (not individual packets). As an example, a MITM attack was started at 748.19 seconds into the data collection, and ended 781.87 seconds in. This was logged with UNIX timestamp, wall clock timestamp and relative time in these two lines:

[1678444922.268 – 2023-03-10 10:42:02 – 748.19] [BAD-MITM-START] Manual MITM starts

[1678444955.948 – 2023-03-10 10:42:35 – 781.87] [BAD-MITM-END] done

Labels for individual packets can be inferred from the entries and timestamps used in the log-file. The network events are described in Section 3.

### 3.2 Data cleaning

To protect Westermo, the data set was analyzed prior to release. Some traffic that was unwanted, or that could possibly reveal details of various Westermo assets, has been removed. In order to prepare the reduced and extended data sets, traffic going to or from the SimFact node was removed. In these analysis and filtration steps, Python3^2^ and Scapy^3^ was used.

### 3.3 Network flows, processed data for machine learning

Instead of analyzing network traffic packet by packet, it is convenient to analyze on a network flow level [6]. A flow can be defined as a set of packets with a common source, target, and protocol that are close in time, or in many other ways. In this data set, we have analyzed the network traffic with the ICSFlowGenerator tool for ICSSIM (ICSFlow)^4^ to get information on flows [2,3]. The tool is implemented in Python with the Scapy library. It iterates through the raw PCAP data and creates CSV files with flow features. Here, a flow is defined as having a common source, destination, protocol and that come in a tight interval of time (500 ms^5^). Network flows typically consist of packets with a given source and destination address (IP and port) and protocol. However, in our customized concept of network flow, we do not consider the ports, and aggregate packets with the same protocol between two network addresses because our simulation uses Modbus with fixed ports on the server side, whereas clients could use different ports which are now aggregated. Each network flow is characterized by a set of extracted features that can be classified into three categories: flow features, general features, and TCP features. Flow features encompass fundamental attributes such as source and destination addresses, as well as the flow's network protocols. General features provide information about the network traffic within the flow. For instance, they include metrics such as the number of packets sent and received, the size of packets, the length of flow, as well as the average payload of the packets. TCP features, on the other hand, are specifically extracted for TCP flows. These features comprise details about TCP flags, TCP headers, and packet delays. For a comprehensive list of all the extracted features and their explanations, please refer to the reference [2]. The extracted features are stored in CSV files, where each file contains 54 columns. Among these columns, 50 columns represent the various features, 4 columns represent the labels, and there may be some additional metadata columns. An overview of the counts of network flows per node and per data set can be found in Table 2.

**Table 2:** Overview of the NST label distributions of the Network Flows per node for the Reduced and Extended data sets. In bold, the differences between the two data sets on the label distribution.

	Reduced							Extended						
	left		bottom		right		All	left		bottom		right		All
	*N*	*%*	*N*	*%*	*N*	*%*	*N*	*N*	*%*	*N*	*%*	*N*	*%*	*N*
Portscan 1	229	*2.7*	29	*0.1*	9	*0.2*	267	229	*2.7*	29	*0.1*	9	*0.2*	267
Portscan 2	1483	*17.4*	397	*1.1*	299	*6.3*	2179	1483	*17.3*	397	*0.7*	299	*6.3*	2179
Bad SSH	1952	*22.9*	751	*2.1*	265	*5.6*	2968	1952	*22.8*	751	*1.4*	265	*5.6*	2968
Bad IP	313	*3.7*	2103	*5.9*	261	*5.5*	2677	313	*3.7*	**3315**	***6.0***	261	*5.5*	3889
Same IP	367	*4.3*	2453	*6.9*	311	*6.6*	3131	**367**	***4.3***	**3902**	***7.0***	**328**	***6.9***	4611
MITM	102	*1.2*	504	*1.4*	102	*2.2*	708	102	*1.2*	504	*0.9*	102	*2.1*	708
Normal	4087	*47.9*	29167	*82.4*	3473	*73.6*	36727	**4095**	***47.9***	**46527**	***83.9***	**3485**	***73.4***	54107
Total	8533	*100*	35404	*100*	4720	*100*	48657	8555	*100*	55425	*100*	4749	*100*	68729

**Table 3:** Overview of hardware used.

Name	Initial IP	MAC	Hardware
A1	198.18.134.99	00:24:9b:6d:b8:89	Laptop
A2	198.18.134.14	b8:27:eb:d1:b7:ef	Raspberry Pi 3B+
hub	198.18.134.1	00:07:7c:88:6e:83	Westermo router
left	198.18.134.2	00:07:7c:88:6e:63	Westermo router
filler	198.18.134.3	00:07:7c:29:de:41	Westermo router
right	198.18.134.4	00:07:7c:29:de:61	Westermo router
top	198.18.134.5	00:07:7c:8c:43:83	Westermo router
bottom	198.18.134.6	00:07:7c:8c:43:63	Westermo router
PLC1	198.18.134.11	b8:27:eb:6d:4f:4b	Raspberry Pi 3 v1.2
PLC2	198.18.134.12	b8:27:eb:5b:50:19	Raspberry Pi 3B
HMI1	198.18.134.15	b8:27:eb:15:88:9c	Raspberry Pi 3 v1.2
SimFact	198.18.134.31	b8:27:eb:3e:5d:96	Raspberry Pi 2B

In the CSV files, flows have been labeled with two different strategies: Injection Timing (IT) strategy [7] and Network Security Tools (NST) [4]. For the IT strategy, we label all traffic as anomalous during an ongoing attack, whereas the NST strategy only labels traffic from or to the attacker as anomalous. The NST strategy seems to reflect on the events more accurately.

**Fig. 2:**
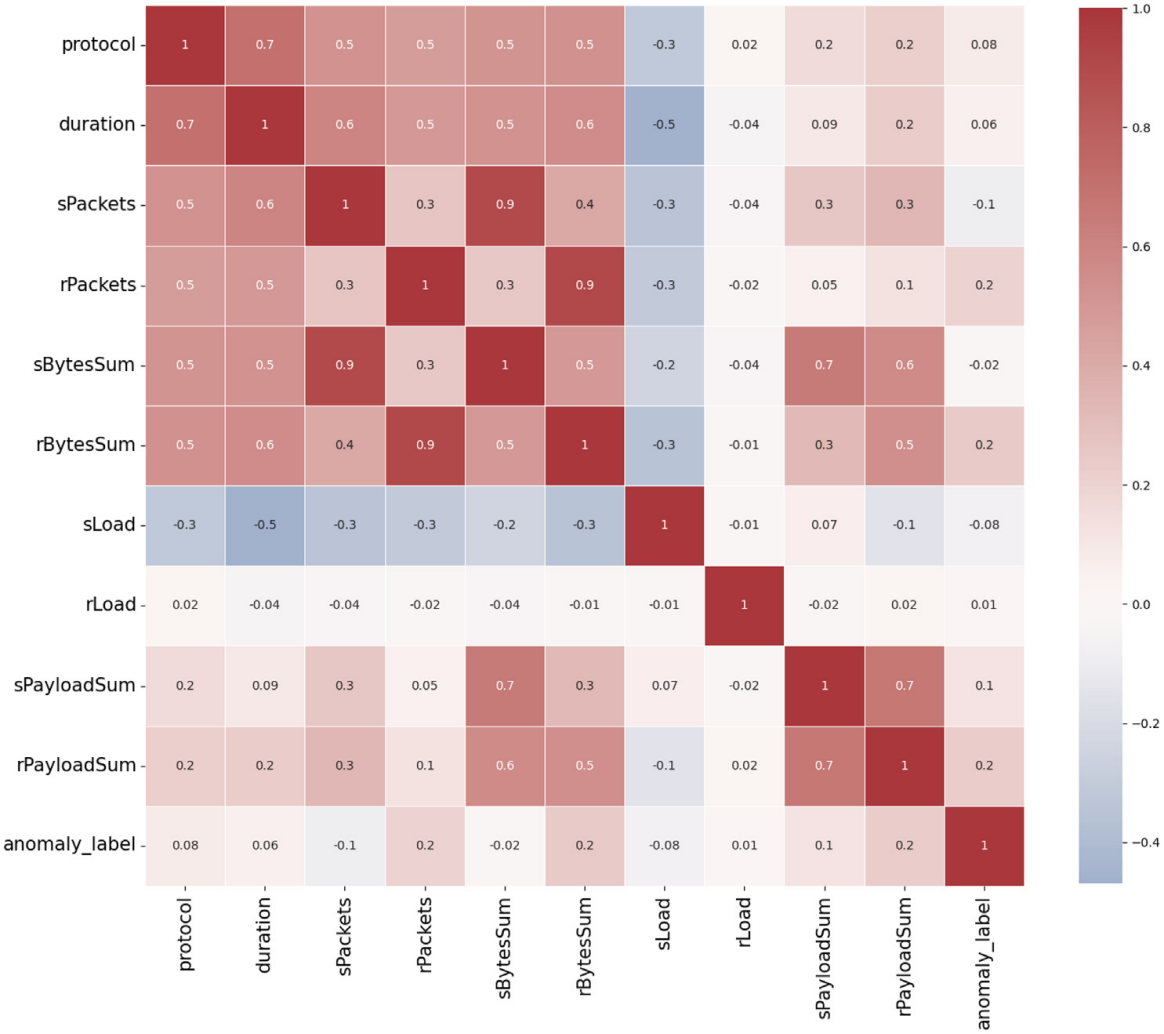
Pearson correlation matrix of the extracted features and the binarized labels (anomaly detection) for the Reduced data set.

The main difference between the Reduced and the Extended data sets concerns the network flows annotated as normal and as misconfigurations. The data is imbalanced where 75% of the whole traffic is labelled as normal, 18.5% for misconfigurations (and bad SSH), and 6.5% for the attacks (portscan and man in the middle) as can be seen in Table 2, the protocol distribution of the network flows for each node is represented. The most frequent protocol is TCP used in 70% of the cases and massively in the bottom node due to the PLC-to-PLC traffic, see Fig. 4. Address Resolution Protocol (ARP) is the second most important protocol (18% of the total) from the data set equally employed by each node. Other protocols appear to a lesser extent. Concerning the extracted features for describing the network flows, a correlation heatmap is shown in Fig. 2. The Pearson pairwise correlation coefficient is computed for the most representative features. Features are also compared with a binary representation of the traffic label (normal or anomaly). It is interesting to notice the highest correlation coefficient between features and the binary label is around 0.2 showing a relatively small linear correlation. This may suggest that non-linear correlation exists.

**Fig. 3:**
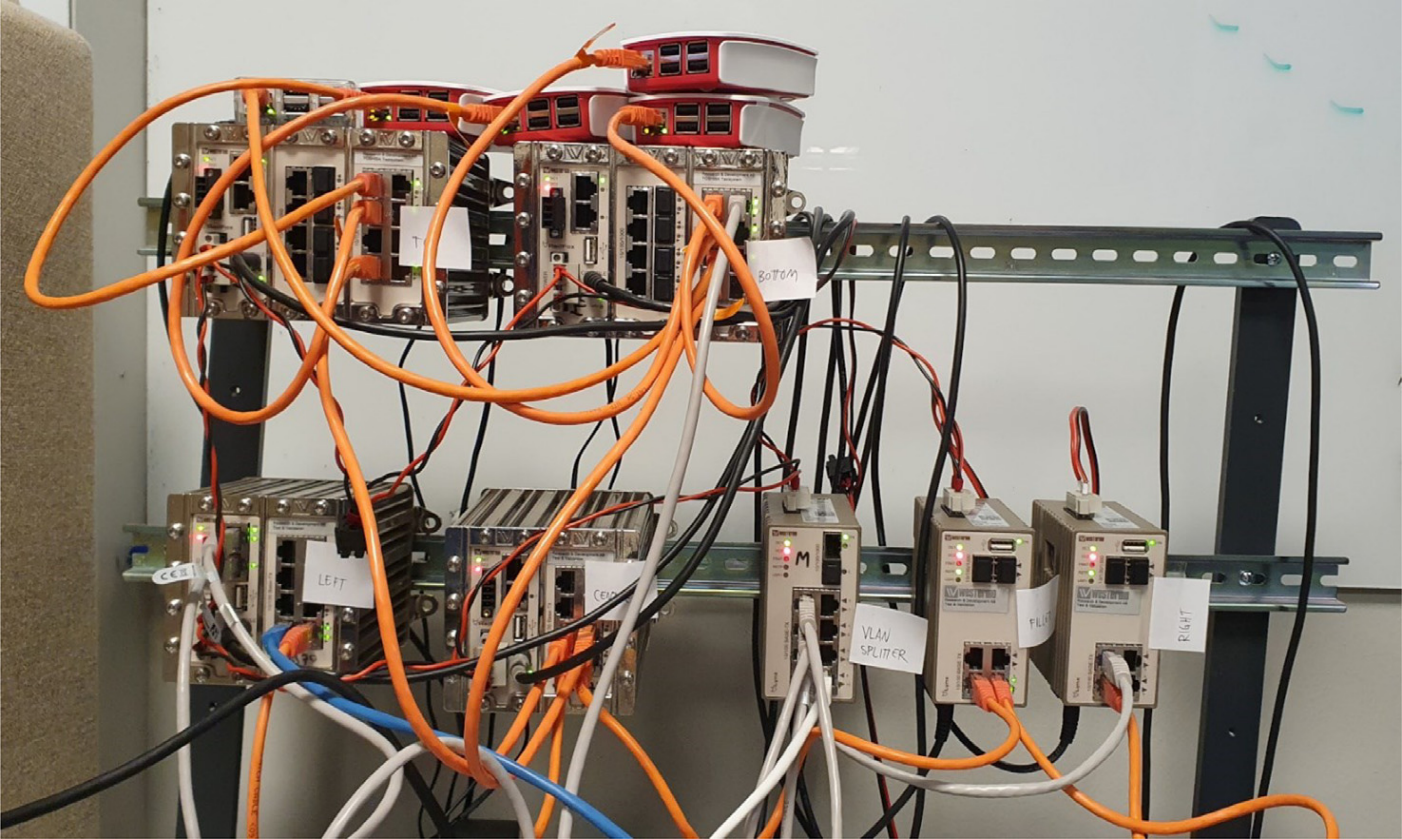
Photo of network topology used during data collection. See Table 3 for details.

**Fig. 4:**
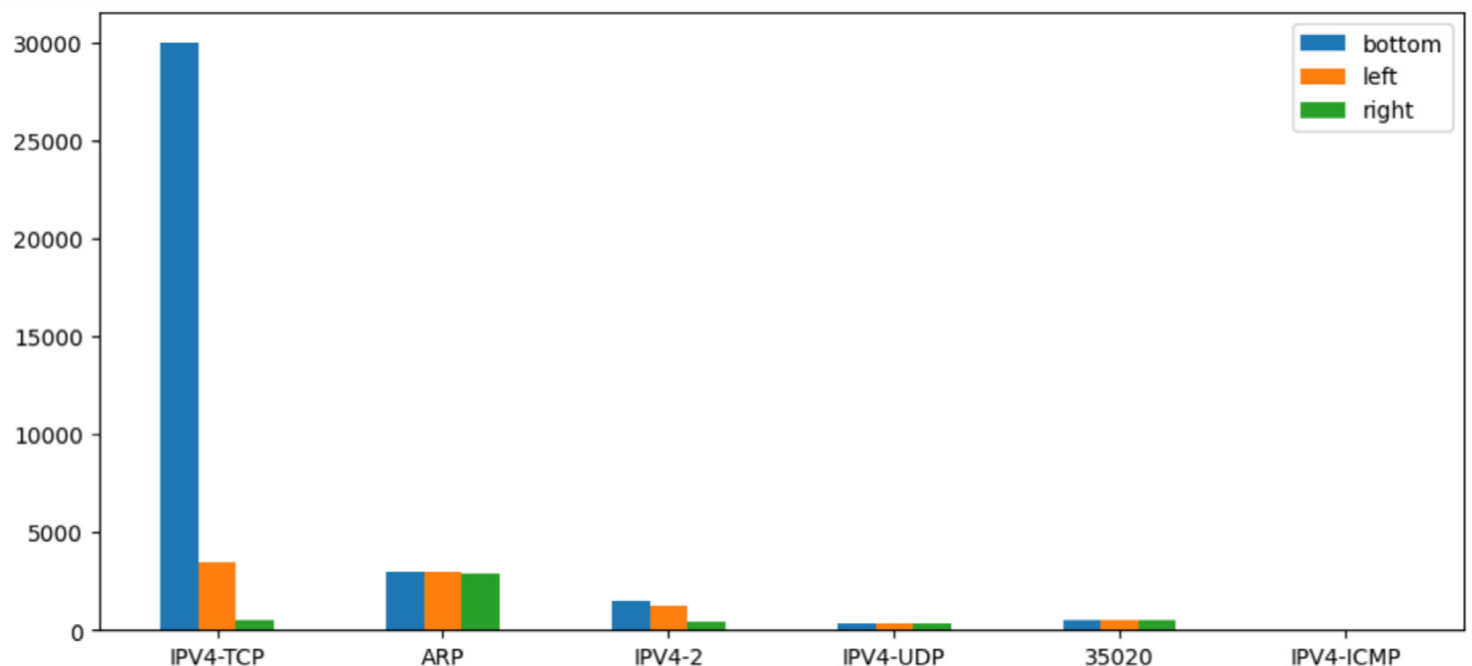
Packet distributions on each node for the Reduced data set when removing RSTP packets.

## 4 Experimental Design, Materials and Methods

As described in Section 2, the network traffic was collected in a physical network topology constructed to be similar to an industrial communication network, while simulating a bottle filling factory.

To trigger network events, node A1 ran scripts implemented with Westermo's test automation framework [10]. Node A2 was used for the man-in-the-middle (MITM). The network events were:

1. Good SSH: a user uses the secure shell protocol (SSH) to login to one of the devices and checks the contents of a log file. This was implemented by adding the user *`alice’* with password *`HarryPotter123’* on the devices, and then logging in from A1, checking the log file, and then logging out.
2. Bad SSH: attacker A1 creates many unsuccessful SSH login attempts in parallel. This was implemented by selecting a random router as target, and generating a random number of usernames and passwords based on the Mirai botnet^6^. The login attempts were conducted in parallel threads.
3. Misconfigured IP: A user sets an invalid IP on a router, e.g., setting it to 198.134.18.33 instead of 198.18.134.33 (note the swapped second and third octet). After some time, a reasonable address is configured. This was configured over a serial console to the router (i.e., the configuration cannot be discovered in the network data, only the effect of the configuration) using already existing functionality in Westermo's test framework.
4. Misconf same IP: A user sets the same IP on two devices, again, by using a serial connection not affecting the network. After some time, a different address is set. Again, this was configured from the console.
5. Port scan: A1 runs the network mapper (nmap)^7^ to scan the ports of one or more devices in the network.
6. MITM attack: A2 runs a MITM attack, steals network traffic in a link, and rewrites certain packets. This event was conducted using the attack toolbox from ICSSIM.

The events were conducted in batches, one after the other, and repeated 16 times for a total of 96 events. Between each event, there were between 12 and 28 seconds of time for recovery and for the network to be idle. This recovery time was more than enough, which was indicated by the normal operation of the HMI between events. Each batch had the order of the events randomized. The total duration of the data recording was about 5440 seconds, or a little over 90 minutes.

## Ethics Statements

To protect Westermo and individuals that contributed to the data, information security risk workshops have been conducted at Westermo. Data was collected with the permission from Westermo. This paper does not contain any studies with human or animal subjects by any of the authors.

## CRediT authorship contribution statement

**Per Erik Strandberg:** Conceptualization, Methodology, Software, Writing – original draft, Writing – review & editing, Supervision. **David Söderman:** Methodology, Software, Writing – review & editing. **Alireza Dehlaghi-Ghadim:** Methodology, Software, Validation, Writing – review & editing. **Miguel Leon:** Validation, Writing – review & editing. **Tijana Markovic:** Validation, Writing – review & editing. **Sasikumar Punnekkat:** Conceptualization, Writing – review & editing, Supervision. **Mahshid Helali Moghadam:** Writing – review & editing, Supervision. **David Buffoni:** Validation, Writing – review & editing.

## Data Availability

Westermo network traffic dataset (Original data) (github).

## Appendix Supplementary materials

Image, application 1

## Footnotes

6 https://github.com/jgamblin/Mirai-Source-Code, see mirai/bot/scanner.c line 123.

7 https://nmap.org/.

## Footnotes

1 https://github.com/AlirezaDehlaghi/ICSSIM.

2 https://www.python.org/.

3 https://scapy.net/.

4 https://github.com/AlirezaDehlaghi/ICSFlow.

5 The interval of 500 ms was selected because it is the logic loop period of the ICSSIM components. Users of the data set could experiment with other periods using the raw data and ICSFlowGenerator.
