# Supplementary figures and images for: The Westermo network traffic data set
Source: Data Brief. 2023 Aug 24;50:109512. doi: 10.1016/j.dib.2023.109512 (PMC10482735; doi:10.1016/j.dib.2023.109512)

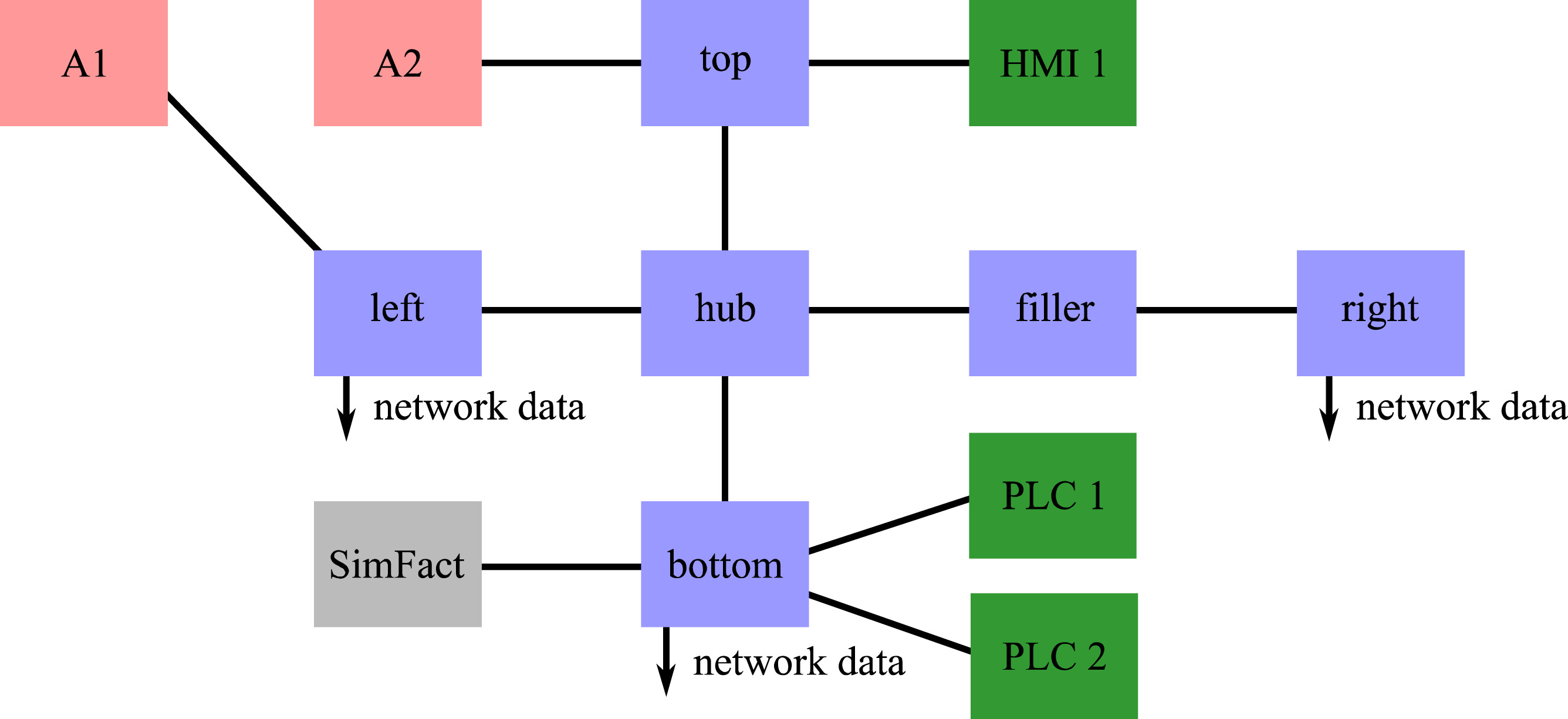

Supplement: Supplementary file 1 [file mmc1.jpg]
